# Effect of alternate nostril breathing on evoked potentials among internet addicts

**DOI:** 10.6026/973206300181075

**Published:** 2022-11-30

**Authors:** Karthika Priyadharshini Udayakumar, Pavithra Mahendiran, Jayamala Annachira Kushalappa, S Murugaiyan

**Affiliations:** 1 Department of Physiology, Sri Venkateshwaraa Medical College Hospital and Research Center, Ariyur, Pondicherry, India; 2 CRRI, Sri Venkateshwaraa Medical College Hospital, and Research Center, Ariyur, Pondicherry, India; 3 The World Community Service Center, Puducherry, India

**Keywords:** Alternate nostril breathing, internet addiction, p300, visual evoked potential

## Abstract

Increased availability of smartphones and easy access to the internet among adolescents has resulted in Internet Addiction (IA). Effects of IA and Pranayama on evoked potential are available but studies on the comparison of immediate and 6-week effects of alternate nostril breathing on evoked potentials among medicos with internet addiction are not available as per our search in PubMed, hence the study was chosen. In this comparative study 100 male and female medical students aged between 18-25 years, with internet addiction scores ≥ 50 were included as study participants. P300 auditory event-related potential and pattern reversal visual evoked potential (VEP) were recorded before, immediately, and 6 weeks after practicing pranayama. Repeated measure ANOVA shows statistically significant change (P<0.05) in P300 amplitude, P100 latency, N145 latency, and VEP amplitude. The post hoc Bonferroni test shows that P100 latency and N145 latency significantly reduced immediately after 15 minutes of pranayama. P300 amplitude and VEP amplitude significantly increased only after practicing pranayama for 6 weeks. Pranayama has an immediate effect on latency, but it takes 6 weeks of practice to significantly change amplitude.

## Background:

The Internet has become an unlimited space, for information exchange, social networking, and the development of cyber behaviors [1]. Behavioral addiction is a compulsion to repeatedly engage in action until it causes negative consequences to a person's physical, mental and social well-being. Behavior persisting, despite these consequences, can be taken as a sign of addiction [2]. Internet use has been linked with many benefits to students, for improving their academic knowledge, and research skills, and for completion of assignments, but there has been a growing concern regarding the risk associated with excessive use of the internet. Internet addiction (IA) has become a serious behavioral health problem in Asian countries [1]. New technologies make our daily lives easier but children and young adults are bound to misuse and overuse technology through computers and smartphones [3]. Since adolescent age groups are psychologically immature and are in a critical period of behavioral change, they constitute the potential risk group for IA [4]. They are also prone to a sedentary life and obesity [5]. Various studies have also shown that IA was associated with mental health problems like low self-esteem, stress, anxiety, poor sleep quality, suicidal tendency, and academic problems [6-7]. It was found that 17.4% of medical students were found to be addicted to the internet with an IAT score > 50 [8]. IA is described as the inability to stop internet overuse, a tendency to perceive offline time as meaningless, excessive irritation and aggression during deprivation, and a psychiatric condition involving maladaptive ideas and pathological behaviors [9]. For Internet addicts, the network information that causes their addiction behaviors mainly comes from the visual channel input (sensory channel), and most of the time they spend their time in front of the screen. Ding W N et al., reported altered visual and auditory function on chronic exposure to online game stimuli (a component of internet addiction) [10]. Visual evoked Potential (VEP) is a more sensitive electrophysiological test for assessing optic nerve dysfunction. Evoked potentials are mostly used to study clinical disorders related to the central nervous system [11]. Internet-addicted individuals were found to have modulation in the auditory and visual event-related potentials. These victims were also associated with cognitive decline [1^2^]. P300 auditory event-related potential is a subjective measure indicating attention, information processing, and short-term memory [13]. P300 latency is an index of stimulus processing and a motor-free measure of cognitive function [14]. The grey matter volume of brain regions, related to sensorimotor process and cognitive control was found to be reduced with an internet addiction score > 50 [15]. Internet gaming disorder was found to reduce the amplitude of P300 and increase the latency of P300 [16]. Impulsivity, deficiencies in executive function, and working memory were all found to be associated with Internet addiction [17-18]. Yoga and meditation can be used therapeutically in the treatment of stress, anxiety, depression, and other psychiatric addictive disorders like IA, as it increases self-confidence, mind relaxation, attentiveness, and decreases irritability [19,20]. According to yoga, Alternate nostril breathing (ANB) is a technique of controlled breathing on right and left sides of the nose which are in connection with the opposite half of the cerebral hemisphere [21]. Cardiorespiratory parameters, evoked potentials, and cognition were known to get altered immediately after practicing pranayama [14, 22]. Telles et al. have reported that there is an increase in P300 amplitude and a decrease in latency after practicing pranayama for 40 minutes [23]. Pranayama is known to relieve stress by modulating autonomic activity [24]. Takroo et al. have reported decreased P100 amplitude after practicing asanas and pranayama for 2 weeks [25]. However, the immediate and 6 weeks effect of ANB on P300 and VEP among internet addicts were not studied widely therefore, it is of interest to compare the immediate and 6 weeks effect of ANB on P300 and VEP among medicos with internet addiction. The objectives were to record VEP and P300 among medicos with internet addiction scores≥ 50 and to compare all the parameters before, immediately, and 6 weeks after practicing pranayama.

##  Materials & Methods:

##  Selection of study participants based on inclusion and exclusion criteria:

This comparative study was conducted in the Research lab of the Department of Physiology, after obtaining the Scientific Research Committee (Ref: SVMC/SRC/2019/12/CTR404 dated 14.2.2019) and Institutional Ethics Committee clearance (Ref: SVMCH/IEC/2019-Feb/8 dated 20.3.2019). The estimated sample size was 93 with an 80% Confidence Interval using Open Epi software based on the 17.4% prevalence of Internet addiction among MBBS phase II to Final MBBS medical students [8].Considering the non-responsive rate and few losses to follow-up, a total of 100 participants were included in the study after obtaining informed consent. The age of study participants ranges from 18-25 years with a normal BMI of 18-24.99 kg/m2 and an internet addiction test (IAT) score ≥ 50 [26].The study was conducted between July 2021 to September 2021. Subjects with a known history of neurological/psychiatric illness, person on chronic medication, sports person or those who regularly practice yoga, known cases of systemic disease like hypertension, or diabetes, those who are known to have a refractive error and other ophthalmological disorders, history of respiratory ailments including nasopharyngeal obstruction, external ear obstruction or known case of hearing disorder, history of chronic smoking, alcoholism were excluded from the study. All the participants were asked to fill out the self-constructed questionnaire on internet use information to gain information on the duration of internet usage on weekends and weekdays, gadgets used, and the purpose of using the internet. Internet Addiction test questionnaire created by Kimberly Young, a globally accepted tool for assessment of internet addiction was used to assess the internet addiction (after obtaining permission to use the questionnaire from Stoelting-Psychology). It consists of 20 items rated on a 5-point Likert scale which takes 5 minutes to complete. A total score of 0 to 30 is considered a normal Internet usage level; scores of 31 to 49 indicate mild Internet addiction; 50 to 79 reflect moderate Internet addiction, and scores of 80 to 100 indicate severe dependence [27]. Medicos with moderate to severe internet addiction with scores ≥ 50 were included as study participants. Participants were asked to avoid intake of substances like tea and coffee 2 hours before the test. After explaining the procedure and clarifying the doubts, participants’ anxiousness was relieved before recording the parameters. P300 auditory event-related potential and visual evoked potential were recorded in the following method in the research lab, under the ambient temperature of 20-25°C from 4.00 to 5.00 pm. 

## P300–auditory event-related potential:

Event-related Potential (ERP) recordings from the scalp of the patients were done in a standard audiometric soundproof room using Neurostim NS2, Medicaid system, Chandigarh. These potentials were recorded using Ag/Ag Cl cup electrodes fixed with EEG paste, from standard locations using a 10-20 International system. After cleaning the site with spirit and cotton, the electrodes were placed at Cz (active electrodes at the vertex), FPz (ground electrode on the forehead), and A1, A2 (reference electrode on the ear lobules). The recordings were obtained in response to the standard auditory "Odd-ball paradigm". The positive wave P300 occurs if the subject is actively engaged in the task of detecting the target stimuli occurring along with the standard stimuli at 300 ms. The skin electrode contact impedance is kept below 5 kilo ohms (5KΩ). During the recording session, subjects were instructed to fix their eyes on a particular spot on the wall in front to avoid electro-oculographic artifacts due to eye movements. P300 latency in milliseconds (ms) and peak amplitude of N2 toP300 in microvolt (µV) were evaluated [13-14].

## Visual evoked potential:

VEP was recorded using Neurostim NS2, Medicaid systems, Chandigarh. Visual acuity and field of vision of all the subjects were assessed before the recording. Each subject was seated at a distance of 1 meter from the pattern generator screen in a dark air-conditioned room and was asked to look at the central spot on the screen with one eye, the other being patched. Thus, the recording was done in each eye separately. The pattern shift stimulus on the TV monitor is white and black checks of 15x15 mm size. The scalpel electrode was placed according to the 10-20 international system. The active electrode was placed at O_z_, which is the highest point in the occiput. The reference electrode was placed at Fpz, which is 12 cm above the nasion. The VEP_s_ are picked up as the difference between active and reference electrodes. The ground electrode was fixed at the wrist. The electrode impedance is kept below 5KΩ, with automatic artifact rejection. The latencies of N75, P100, N145 in ms, and N75-P100 amplitude in µV were analyzed [28].

## Alternate nostril breathing:

The participants were made to sit comfortably with the head, neck, and trunk erect, in a calm room. They were instructed to close the right nostril with the right thumb using Vishnu mudra and to exhale completely through the left nostril without exertion and jerkiness under the supervision of the yoga instructor. At the end of the exhalation, the participants were asked to inhale through the left nostril and exhale slowly and completely through the right nostril. In the next cycle, they were made to inhale through the right nostril (keeping the left nostril closed), following which exhalation through the left nostril. This was made to practice for about 15 min,5 days a week for about 6 weeks.[21]. P300 and VEP were recorded immediately after and at the end of 6 weeks.

##  Results:

The normality was tested using the Kolmogorov-Smirnov test. Dataare tabulated as Mean±SD. Repeated measure ANOVA was used to compare before, immediately after, after and 6 weeks of practicing pranayama. The post hoc Bonferroni test was used for intergroup comparison. It was found that all the participants (58 Female and 42 Male) owned a smartphone, in addition to that 6.25% of them were using a desktop at home, 1.38% was using a tablet and 1.38% was using an iPad as a means of internet use. The mean hours spent on the internet on weekdays (Monday to Friday) were 20.59 hours and 10.26 hours on weekends (Saturday & Sunday). Around 80% of the participants were found to spend their online time in social networking and playing games and the remaining were using the internet for watching videos, surfing, texting, and downloading. Table 1 shows the mean age, height, weight, and BMI (Body Mass Index) of the participants. All the participants were within normal BMI. The mean Internet addiction score is 63.52±12.32, indicating all the participants belong to the moderate to severe IA Category.

## Discussion:

There is a significant increase in P300, and N75-P100 amplitude after 6 weeks of practicing pranayama however latency of P100, and N145 significantly reduced immediately after 15 minutes of pranayama. Speed of identification of the stimulus, memory, and decision-making process affect the P300 latency while receptiveness and attentiveness affect the P300 amplitude [29]. Higher mental performance produces shorter latencies. Greater attention produces larger P300 waves (increases the amplitude) [30]. Latency and amplitude of P300 in the table: 2 show that mental attention, stimulus processing speed, and efficiency increase after the practice of pranayama similar to a study done by Telles et al. [23]. This is due to reduced sympathetic activity and shunted autonomic arousal resulting in mental relaxation and improved attention upon practicing pranayama [31]. Stimulation of the Vagus nerve following controlled breathing was found to improve cognition by promoting neural plasticity and release of neurotransmitters like acetylcholine, epinephrine, and brain-derived neurotrophic factor (BDNF) [32]. According to Bhavanani et al. inhibition of ascending reticular activating system following pranayama could have resulted in enhanced neural processing [33]. Kyizom et al. have reported that alleviation of stress, up-regulation of hippocampal 5HT1A receptors, and decrease in cortisol levels upon practicing pranayama might be the reason for increased P300 amplitude and decreased latency among internet addicts [13]. Hariprasad et al. have reported that an increase in the volume of the bilateral hippocampus is a potential mechanism for enhancing cognitive function in practicing yoga [34]. Our results are similar to the study done by Sharma VK et al. where 12 weeks of pranayama practice improved cognition [35]. VEP parameters in (Table: 2see-PDF) show that there is a significant decrease in P100, and N145 latency, and an increase in N75-P100 amplitude after pranayama. Our results are similar to a study done by Biswas et al. where the Deep breathing technique was found to decrease the P100 latency and increase the N75-P100 amplitude among people with migraine [36]. Studies on rats reported that stress was known to prolong the latency and decrease the amplitude of VEP parameters [37]. Alleviation of stress following pranayama might be the reason for significant change in P100 latency and VEP amplitude in our study. In contrast to our result, Trakroo et al. have reported that there was a decrease in the amplitude and prolongation of latency of P100 after practicing pranayama for 6 months [25]. Hence from this study, we found that latency shortened immediately after practicing pranayama whereas amplitude increases with regular practice of pranayama for 6 weeks since it requires the recruitment of nerve fibers. Participants were advised to undergo regular counseling sessions with psychologists in our college to overcome addictive behavior similar to that of service for healthy use of technology undertaken at NIMHANS, Bangalore [38]. Psychiatrists of our college addressed MBBS students on healthy usage of technology and during the same session, role plays were conducted for the students on boons and curses of technology. After the completion of this study, the following measures were implemented in our institute. As a general rule usage of mobile phones is restricted for studeons in our campus. Students were made to involve themselves in yoga and other forms of physical activity.

## Conclusion:

The immediate effect of pranayama increases the speed of stimulus processing and conduction velocity as indicated by a significant reduction in latency. However, 6 weeks of practice is required to improve the amplitude of P300 and VEP among internet addicts. Hence the regular practice of pranayama can be advised to improve cognitive and visual processing. Thus, the hypothesis stating ANB improves the P300 and VEP parameters among the participants with internet addiction was proved.

## Strength of the study:

The main strength of our study is the use of an objective test (P300) for the measurement of cognition rather than a subjective test which could have caused bias. Since the students were made to practice pranayama under the supervision of a certified yoga master, there were no dropouts.

## Limitations:

Limitations of our study are the small sample, and the low level of evidence being a non-randomized interventional study. The follow-up internet addiction score at the end of 6 weeks of pranayama and after counseling sessions were not measured. Though strict inclusion and exclusion criteria were followed, other forms of lifestyle activities among the participants were not controlled.
